# A holistic decision-making approach for identifying influential parameters affecting sustainable production process of canola bast fibres and predicting end-use textile choice using principal component analysis (PCA)

**DOI:** 10.1016/j.heliyon.2021.e06235

**Published:** 2021-02-17

**Authors:** Ikra Iftekhar Shuvo

**Affiliations:** University of Alberta, Canada

**Keywords:** Canola, Bast fibre, Polymer, Cultivars, Textile properties, Sustainability, Principal component analysis (PCA), Retting, Enzyme, Chemical treatment

## Abstract

Recent research has discovered and validated that canola fibre polymer has a lower density than major industrial fibres like cotton, jute, hemp, or flax. A few studies have identified key backgrounds that relate to canola fibre polymer production parameters; however, none have modelled an analytical hierarchy process to identify the influential parameters while producing the canola fibre polymers. The current study used Plackett-Burman design analysis to optimize the fibre polymer yield (%) during retting Statistical tools including Fisher's LSD, ANOVA, Pearson's correlation coefficient, and principal component analysis (PCA) were applied for a comparative analysis among four different canola cultivars (HYHEAR 1, Topas, 5440, 45H29). Physical testing and non-parametric statistical analysis tools like Chi-square (X^2^) test were used to investigate the effect of cultivar on the physique of the stems--the source of biomass. This holistic approach was taken to correlate key factors for the sustainable manufacturing of canola fibre polymers. Such knowledge will lay an effective foundation for future material-science research works, consumer wearable manufacturing industries, and engineering design for composite or nonwoven fabrication using this lightweight natural fibre polymer.

## 1 Introduction

The synergy among climate change, rapid deforestation, and rising anthropogenic pollution will have a profound global effect on natural fibre industries, because of their dependence on natural resources. Amidst all the commotion like rising global temperatures, reduction of clean water, losses of agricultural land to rapid urbanization, and quickly-declining biodiversity, the commercialization of new generation natural fibres, particularly canola (*Brassica napus* L.) biomass, has failed to attract mainstream attention. Nature, in its abundance, offers a large amount of canola biomass, which finds no productive or industrial application after oil seed extraction but still can be a sustainable source of cellulosic bast-fibre [1]. Pollution of fresh water sources from rapid urbanization, domestic sewage, industrialization, and rising population has led to the Global Risk Report by World Economic Forum (WEF) to rank the freshwater crisis as the highest risk factor for global population for its deep-lasting and widespread impact [2, 3]. Since canola bast-fibre is extracted from canola plant biomass, almost no water is required for producing this raw material [4]. On the other hand, commercial fibre like cotton requires 550–950 L/m^2^ of water during cultivation [5]. Hence, bast-fibre canola can be both a sustainable and a water-saving source of cellulosic fibres: neither additional irrigation water, nor additional agricultural land is needed for its cultivation. Previous research has revealed that canola fibre is the world's most light-weight bast-fibre due to its intrinsic low density compared to hemp, flax, jute and cotton [4]. Therefore, for sustainability and economy of production, natural fibres produced from canola biomass should be rigorously promoted by countries who are the biggest world producers of canola: Canada (21.3 x 10^6^ million tons/year), China (14.55 x 10^6^ million tons/year), and EU (20.54 x 10^6^ million tons/year) [6]. Stakeholders and policy makers from these developed countries should promote canola fibres as a renewable fibre source [7].

Fibre yield (%) and retting time differ among canola cultivars [4, 8]. Such variations are also predominant in major industrial fibres like cotton, hemp, and flax [9, 10, 11]. Furthermore, plant sizes offer important analytics like fibre content and retting losses [12]; therefore, if the plants are affected by bio-stresses, the biomass production rate will be lowered [13]. For the material industries to get a wider view of biomass of canola cultivars, a comparative study is outlined in this research work. Four different canola cultivars (HYHEAR 1, Topas, 5440, 45H29) were germinated, cultivated, and harvested inside the Crop Technology Centre (CTC), University of Manitoba to investigate the effect of different cultivars on the physique of the stems i.e., the source of the canola biomass for fibre production.

Retting extracts the bast fibres from the canola biomass. A very recent work has investigated the effect of different parameters for mechanical extraction of bast fibres using retted stems, but did not conduct any work on retting bath parameters, such as temperature, material to liquor ratio (M:L), and stem orientation [14]. No analytics in any previous works comprehensively and statistically displayed the impact of different retting bath parameters on optimizing the fibre yield (%). Therefore, using the Plackett-Burman design model, a ruggedness test was conducted in this research to statistically analyze the most influential parameters to obtain optimum fibre yield (%) while modelling the retting bath parameters for extracting bast fibres from canola biomass. Further, factorial ANOVA was introduced to analyze the main effects and interaction level of two major canola fibre properties (fibre softness and length) following chemical and enzymatic surface modification of the four different canola cultivars (HYHEAR 1, Topas, 5440, 45H29).

Virgin canola fibres can be used for producing nonwovens, whereas modified (chemical or enzyme treated) fibres were found suitable for textile application as mentioned previously [1]. This current work demonstrates a comparative study between the mechanical properties of virgin canola fibres and chemically treated canola fibres and investigates the correlation co-efficient [4]. Understanding the mechanical behavior of virgin canola fibre is necessary for design of nonwoven fabrication to be used in industrial use or composite application. Hence, breaking load, strength index, breaking tenacity, and tensile strength of the four different cultivars of virgin canola fibres were statistically investigated in this study to facilitate the choice of the appropriate canola cultivars based on end use requirements.

**Figure 1:**
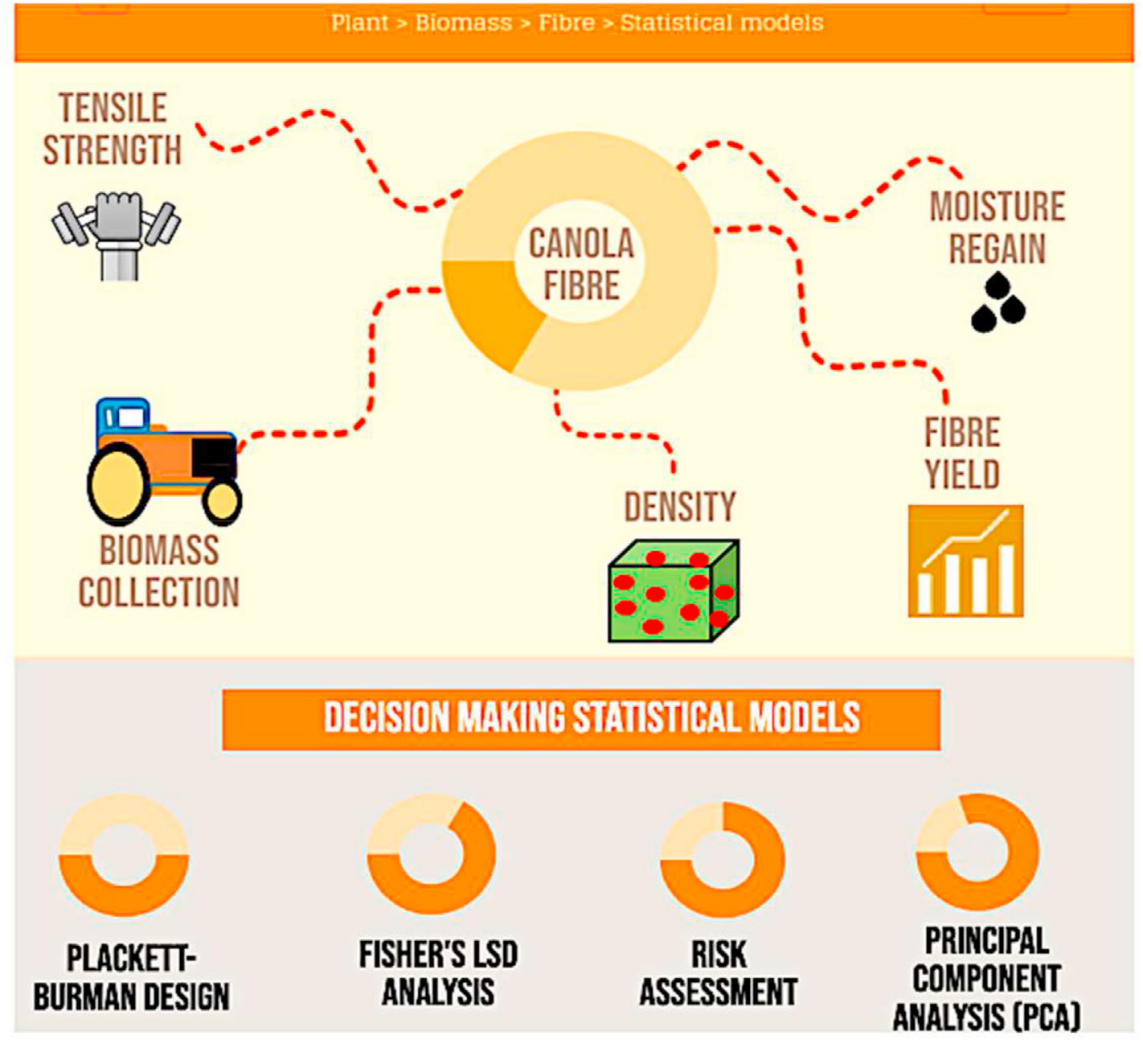
Visual abstract of the current study for the holistic decision support framework supported by different statistical models.

Finally, PCA (Principal Component Analysis) analytics were implemented in this research work. PCA has been widely used to cluster specimens of similar behavioral patterns based on multiple variables displayed by the specimens [15, 16]. PCA is a novel and an effective multivariate statistical technique that extracts key information from a large group of parameters and data-set to best fit the internal parameters in a way which effectively explains the variance of that data-set. Principal components (PC1 and PC2 combined) can successfully detail 80% of the variance of the data-set, which can decrease to 70% for ecological observations due to inherent variability [16]. PCA is used in a wide spectrum of industries for different purposes, including process yield prediction of protein-A chromatography in bioengineering, identifying critical pollutants and their sources in environmental monitoring system, minimizing information redundancy in civil engineering, predicting the effects of drugs on behavioral brain research, and so on [15, 16, 17, 18, 19]. This novel multivariate statistical tool offers the reliability to formulate an ideal point that will represent an industry grade natural fibre where all the fibre characteristics are within prescribed standard limits set by material scientists or engineers. Therefore, the cluster of specimens or a single specimen from the four different experimental cultivars can be categorized for consumer wearable, nonwoven or eco-composites application as implemented in this current study. Hence, an effort was undertaken to conduct a holistic decision support framework (Figure 1) to reduce the uncertainty of fuzziness and randomness of different variables by statistically analyzing different parameters of canola cultivars to make the sustainable manufacturing process less cumbersome.

## 2 Materials

### 2.1 Plant materials

One commercially grown canola cultivar (Topas) and three experimental cultivars of canola (HYHEAR 1, 5440, 45H29) were used for this research study. 96 seeds per cultivar were germinated inside a growth chamber (day temperature: 22 °C; night temperature: 17 °C) and then transplanted on day 11 to greenhouse water-benches (temperature: 22–25 °C, humidity 40–50%, daylight per day: 16 h light, 8 h dark) of Crop Technology Centre, University Manitoba. Manual plant hydration continued from day 11 to day 31, until automatic plant hydration started inside the greenhouse controlled by Argus Control System Ltd., Surrey, BC, Canada. The irrigation process continued from day 32 to day 110. Many plants were unable to bear their own weight and were supported by a bamboo stick (lodged) until harvesting was finished on 117^th^ day. Virgin canola fibres were collected from the water retted plant stems detailed in the previous research work [4], followed by virgin fibre characterization, plant stem characterization, and retting experiments at the Fibre Laboratory of University of Manitoba and Fibre City of Composite Innovation Centre (CIC).

### 2.2 Chemicals

Different concentrations of chemicals and enzymes were used in the current research study: (i) pectinase enzyme from *Aspergillus aculeatus* (Sigma-Aldrich, Canada) (4%); (ii) acetic acid CH_3_COOH (Sigma-Aldrich, Canada) (10% and 4%); (iii) alkali NaOH (Sigma-Aldrich, Canada) (5%); (iv) Tubingal 4748 (CHT BEZEMA, Germany) softener (3% and 10%); (v) glycerin (Ricca Chemical Company, USA) as wetting agent (0.01% and 0.5%); and (vi) AATCC 1993 WOB (without optical brightener and without phosphate) standard detergent (Testfabrics, Inc., USA) (0.2%).

## 3 Methods

### 3.1 Characterizing virgin fibres and plant stems of canola

#### 3.1.1 Moisture regain

Moisture regain of stems and water-retted virgin fibres was assessed according to ASTM D2495-07 [20]. The specimens were conditioned inside a humidity chamber to avoid any variations of moisture content according to ASTM D1776/D1776M-16 [21], followed by oven drying at 105 °C for 8 h in an incubator according to ASTM D2495-07 [20] standard. The weight of specimen was assessed at room temperature, and after oven-drying. The difference between these two weights is the weight of moisture inside the specimen. The moisture regain thus was calculated by the following formula (Equation 1):

(1) $$\text{Moisture regain }\left(\text{\%}\right)=\left[100\text{ x }\frac{\text{Weight of moisture in the specimen}}{\text{Weight of oven dried specimen}}\right]\text{\%}$$

#### 3.1.2 Diameter

Water retting of canola stem samples was carried out for fibre extraction. Before starting the water retting of the stems, the diameters (dia) of the plant stems for each cultivar were measured in three regions (top, middle and bottom) using a using a digital Vernier slide caliper. The average diameter for each stem was calculated using the following formula (Equation 2):

(2) $$\text{Stem diameter}=\left[\frac{\text{Dia at top }+\text{ Dia at middle }+\text{ Dia at bottom }}{3}\right]\;mm$$

#### 3.1.3 Thermal resistance

At the department of Biosystems Engineering of University of Manitoba, the thermal resistance of plant stems was assessed using a thermal melting point analyzer (Linkam Scientific Instrument, UK). The melting point analyzer comprises a controllable heating plate and is connected to a color monitor ((Dynax, Model: DX- 22L 150A11, UK) to visualize the changes during the testing. After placing 50 g of specimen on the glass plate of the Linkam analyzer, temperature was gradually increased at 10 ^°^C/min. The change in specimen color can be easily seen both by the naked eye and in the color monitor. This method has been widely followed in thermal resistance characterization of canola, cattail and different bast fibres in recent research works [1, 4, 8, 22].

#### 3.1.4 Inherent color property

LabScan XE Spectrophotometer (Hunterlab, USA) was used for a comparative analysis of the whiteness-blackness, L∗; redness-greenness, a∗; and b∗, yellowness-blueness of the plant stems of the four cultivars. A higher L∗ indicates lightness and lower L∗ value indicates darkness. Similarly, higher a∗ value higher indicates the redness, and lower the greenness and vice-versa for a specimen. Hence, if the value of a∗ is positive then it represents redness and the negative value represents greenness. Similarly, if the value of b∗ is positive, it represents yellowness and the negative value represents blueness.

#### 3.1.5 Plant stem retting

The plant stems were retted in a retting bath using different parameters like temperature, time, and material to liquor ratio (M:L). Fibre yield (%) was calculated using the following formula (Equation 3). The overall retting process, retting end point determination, and overall retting fibre extraction method has been detailed in later segment of this study in Table 1.

(3) $$\text{Fibre yield }\left(\text{\%}\right)=\left[\frac{\text{Weight of oven dried water retted fibres}}{\text{Weight of oven dried unretted stem}}\right]\text{x }100$$

**Table 1:** Optimum fibre yield (%) ruggedness test factors, variable details, units, and levels.

Factor no	Variable detail	Units	Level one (-1)	Level two (+1)
A	Retting bath temperature	^o^C	20	40
B	M:L (material to liquor ratio)	gm/ml	1:100	1:150
C	Retting time	hours	144	360
D	Retting orientation	-	vertical	horizontal
E	Stalk rotation frequency	hour^−1^	1 per 24 h	2 per 24 h
F	Stalk diameter variation	high/low	high	low
G	Stalk length variation	high/low	high	low

#### 3.1.6 Mechanical properties

Mechanical properties, such as breaking load (lb), strength index (lb/mg), breaking tenacity (gram-force/tex), and tensile strength (MPa) of the water-retted virgin fibre were assessed using the exact methodology used in the previous research work to characterize the chemically modified canola fibres (treating the water-retted virgin fibres to individualize and to make more flexible and soften) following the formulas (Equations 4, 5a-5e) [4]. These equations are based on ASTM D1445M-12 [23] standard where a Pressley Fibre Bundle Strength Tester (Model F215, SDL Atlas Instrument, USA) was used to measure the breaking load (lb).

(4) $$\text{Strength index }\left(\frac{\text{lb}}{\text{mg}}\right)=\left[\frac{\text{Breaking load }\left(\text{lb}\right)}{\text{Mass of specimen }\left(\text{mg}\right)}\right]$$

(5a) $$\text{Breaking tenacity for HYHEAR }1\left(\frac{\text{gram}.\text{force}}{\text{tex}}\right)=\left[6.42\text{ x Strength index}\right]$$

(5b) $$\text{Breaking tenacity for Topas }\left(\frac{\text{gram}.\text{force}}{\text{tex}}\right)=\left[6.51\text{ x Strength index}\right]$$

(5c) $$\text{Breaking tenacity for }5440\left(\frac{\text{gram}.\text{force}}{\text{tex}}\right)=\left[6.61\text{ x Strength index}\right]$$

(5d) $$\text{Breaking tenacity for }45\text{H}29\left(\frac{\text{gram}.\text{force}}{\text{tex}}\right)=\left[6.85\text{ x Strength index}\right]$$

(5e) $$\text{Tensile strength }\left(\text{MPa}\right)=\left[9.807\text{ x Fibre density }\left(\frac{\text{g}}{\text{cc}}\right)\text{x Tenacity }\left(\text{gram}.\frac{\text{force}}{\text{tex}}\right)\right]$$

### 3.2 Statistical identification of retting variables for optimum fibre yield (%)

#### 3.2.1 Experimental design to conduct ruggedness test

**Figure 2:**
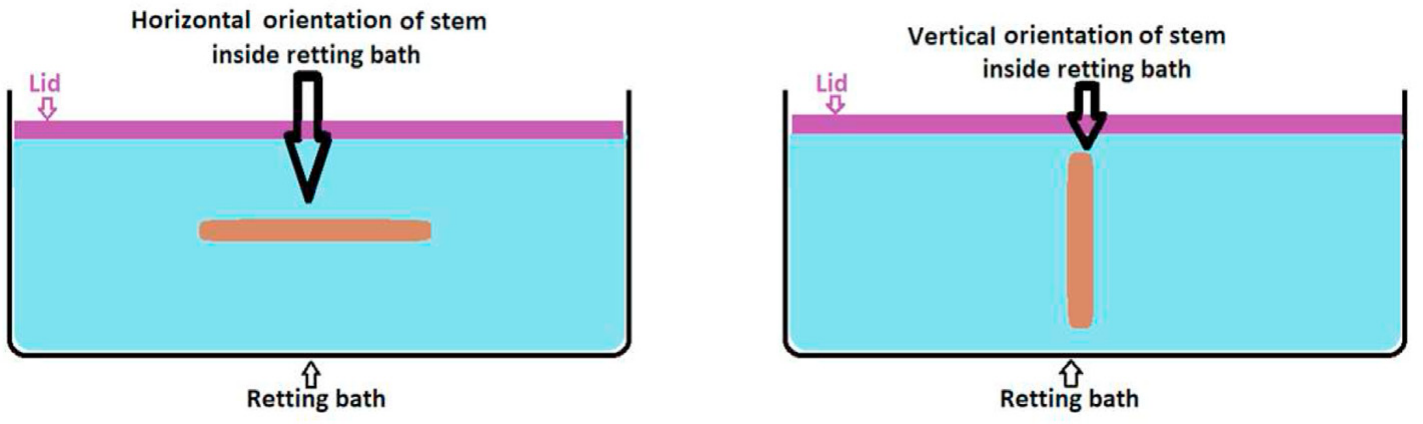
Horizontal and vertical orientation of plant stems inside retting bath.

ASTM E1169-18 standard was used in designing this experimental method to identify the relationship among the influential factors based on the *p*-value (statistically significant at the 5% level) obtained after the two-sided tail probability Student's *t*-test with seven degrees of freedom [24]. The factors of interest in this experimental design are those involved in conducting water retting methods for canola biomass for an effective fibre yield (%). These factors are retting bath temperature (^o^C) [1], material to liquor ratio (gm/ml), retting time (hours), retting orientation (vertical or horizontal) (Figure 2), rotating frequency to prevent over-retting or under-retting [4], stalk (stem) diameter variation, and stalk (stem) length variation (Table 1). The retting method was followed as detailed in a recent research work on canola biomass for producing light-weight bast fibres for technical and engineering applications [4]. The Plackett-Burman design strategy was used to obtain the estimate effect of each factor. Each factor had two alternatives, which were assigned two values, “Level 1” and “Level 2” that are indicated by (-1) and (+1). Categorical factors (non-ordered) are arbitrarily designated “low” or “high” or “-1” or “+1” as per the ASTM E1169-18 ruggedness test standard [24].

#### 3.2.2 Experimental procedures to conduct ruggedness test

The experimental design parameters are illustrated in Table 2. Each experiment was set to run according to the alternative levels outlined in Table 2. Treatment# 1, for instance, displays that canola plant stems were water retted inside a water retting bath at 40 °C (A = +1) for a material to liquor ratio of 1:150 (B = +1) for 360 h (C = +1), where orientation of the plant stems were vertical to the natural water flow (D = -1), plant stems were rotated twice within 24 h (E = +1) (one rotation after each 12 h), and had large stalk diameter (F = -1) and length (G = -1) variations.

**Table 2:** Details of the treatments conducted in this experimental design.

Treatments #	A	B	C	D	E	F	G
1	+1	+1	+1	-1	+1	-1	-1
2	-1	+1	+1	+1	-1	+1	-1
3	-1	-1	+1	+1	+1	-1	+1
4	+1	-1	-1	+1	+1	+1	-1
5	-1	+1	-1	-1	+1	+1	+1
6	+1	-1	+1	-1	-1	+1	+1
7	+1	+1	-1	+1	-1	-1	+1
8	-1	-1	-1	-1	-1	-1	-1

A: retting temperature; B: M:L (material to liquor ration) in retting bath; C: retting time; D: stem orientation during retting; E: Number of times stalk feed has been rotated (i.e., moving the top stems to bottom of the bath and bringing the bottom stems to the top of the bath); F: variation of stalk (stem) diameter; G: variation of stalk (stem) length. See Table 1 for detailed information of each alternative.

For stalk diameter variation (F), the unretted stems were first visually inspected and separated based on three categories: category one (stems with small diameters), category two (stems with medium diameters), and category three (stems with large diameters). To develop high level of stalk diameter variation (F = -1) treatment, category one and category three were combined for sampling preparation and category two was used in low diameter variation (F = +1) treatment [14]. A similar method was followed to develop high (G = -1) and low (G = +1) stalk length variation treatments.

### 3.3 Fibre surface modifications and micrographs by scanning electron microscopy (SEM)

The water-retted virgin canola fibres of HYHEAR 1, Topas, 5440, 45H29, were subjected to four different treatment methods: (i) enzyme [1]; (ii) enhanced enzyme [8]; (iii) 3% softener plus enzyme [1]; and (iv) 10% softener, as demonstrated in a recent research work by the author [4]. SEM microgrpahs were taken for a better view of the virgin fibres prior to surface modification and post modification. SEM micrographs of the fibre-cluster (immediately upon extraction from water retted stems) and a single fibre (after manual individualization) were obtained using a high-resolution FEI Nova NanoSEM (Scanning Electron Microscope) at the Manitoba Institute for Materials (MIM), University of Manitoba. The samples were coated with gold prior to conducting SEM experiments and SEM micrographs were taken at different magnification ranges (100x - 12000x).

#### 3.3.1 Enzyme treatment for surface modification of virgin fibres

Bast cellulosic fibres contain non-cellulosic materials such as lignin and pectin in the interior [25]. These are considered impurities as they make the fibre stiff and hinder their Cotton Spinning Properties (CSP). One-bath pretreatment process of natural cellulosic fibre using an enzyme mixture could produce satisfactory textile properties like excellent water absorbency and high tenacity [26, 27, 28]. Therefore, an attempt was made to investigate the effect of enzyme treatment on water-retted virgin canola bast fibres. The virgin water-retted canola fibre samples from this research work underwent treatment at 40 °C for 90 min using a 200 ml solution containing 4 % Pectinase enzyme from *Aspergillus aculeatus* at pH 5.2, controlled by acetic acid [1]. Following the treatment, the samples were washed with distilled water, dried at room temperature, and oven dried in an incubator for 8 h at 105 °C according to ASTM D2495-07 standard [20].

#### 3.3.2 Enhanced enzyme treatment for surface modification of virgin fibres

This enzymatic surface modification treatment involves three steps as developed by Khan [8], which involves using 0.2 % AATCC 1993 WOB (without optical brightener and without phosphate) standard detergent (Testfabrics, Inc), 0.01% Glycerin (Ricca Chemical Company), 4 % Pectinase enzyme from *Aspergillus aculeatus* (at pH 5.5) followed by incubator drying process according to ASTM D2495-07 standard [20].

#### 3.3.3 3% softener plus enzyme treatment for surface modification of virgin fibres

Recent investigations involving the treatment of bast fibres with enzyme and bleaching agents to improve the CSP properties found that enzyme-treatment reduced the least amount of non-cellulosic material, while bleaching treatment reduced the largest amount of non-cellulosic material [29, 30, 31, 32]. Hence, an attempt was made in this current research work to combine multiple chemical and enzymatic treatment processes on the virgin-retted fibres to improved fibre quality and to achieve the optimum CSP for canola fibres - a four-step canola surface modification model of chemical and enzymatic treatment processes was followed in this current research [1] using 5% NaOH (Sigma-Aldrich Corporation), 0.5% wetting agent (Glycerin), 4 % acetic acid (Sigma-Aldrich Corporation), 3% Tubingal 4748 (CHT BEZEMA) (at pH 4.5), 4 % Pectinase enzyme *Aspergillus aculeatus* (at pH 5.5).

#### 3.3.4 10% softener treatment for surface modification of virgin fibres

The 10% softener treatment process was quite identical to the 3% softener treatment process stated above, except the final treatment process involves treating the acid scoured fibres with 10% Tubingal 4748 at 40 °C for 30 min at pH 4.5 (controlled by acetic acid) developed in our previous research work [4].

### 3.4 Experimental design

**Figure 3:**
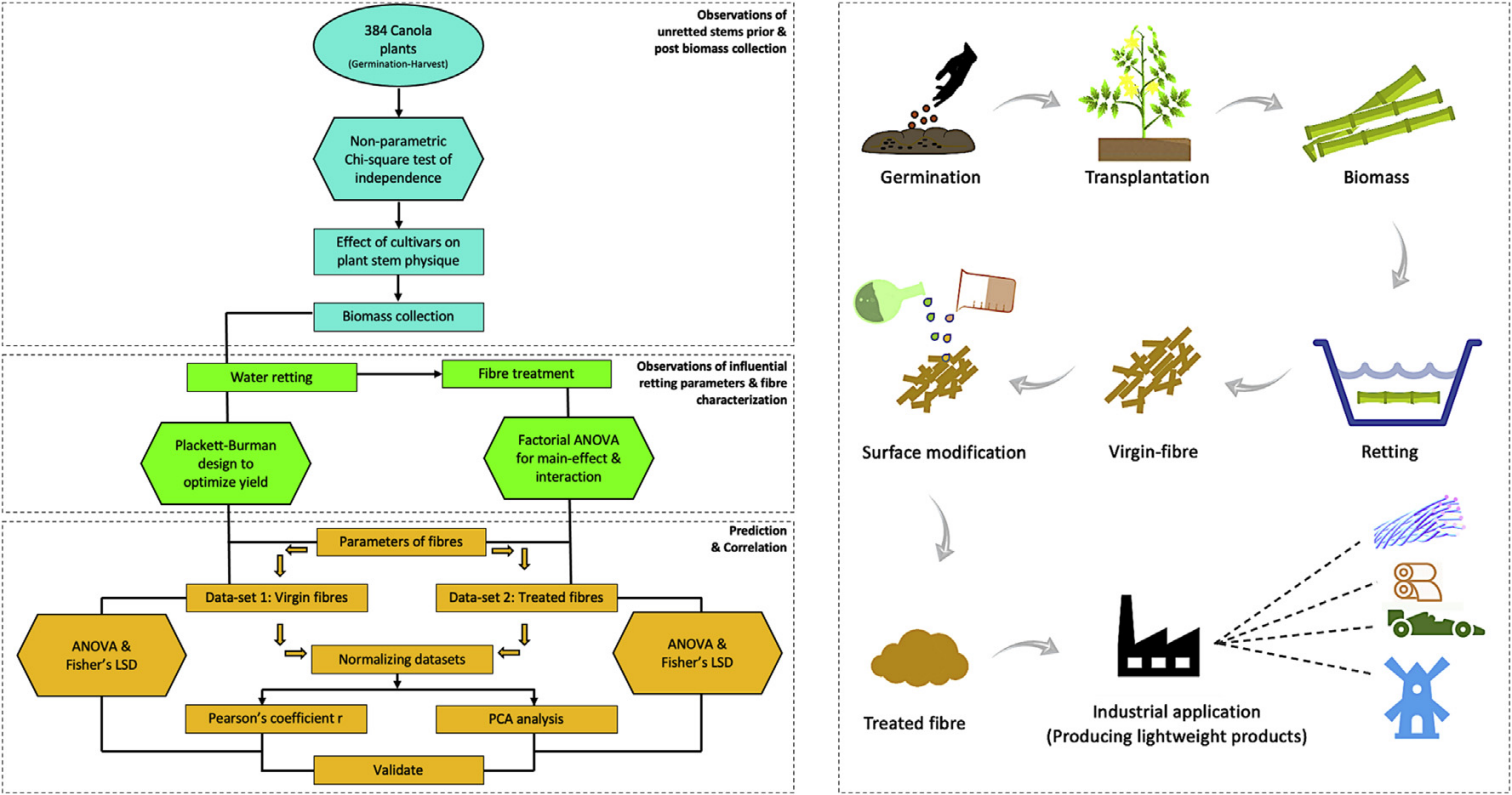
Experimental modelling (left) for a sustainable production process (right) of canola fibre polymer.

This study undertook a holistic approach to detail the challenges and effects of cultivars on each step from plant cultivation to production of a lightweight bast-fibre, as illustrated in the flowchart (Figure 3) representing stepwise methodology and corresponding analytics.

## 4 Results and discussion

### 4.1 Attributes of canola stems prior and post biomass collection

Before discussing observations of canola plant stems, the main source of the canola biomass, it may be effective to discuss briefly the lifecycle of a canola plant for a better understanding of the effect of cultivars on plant stem physique.

#### 4.1.1 Canola growth and development stages

**Figure 4:**
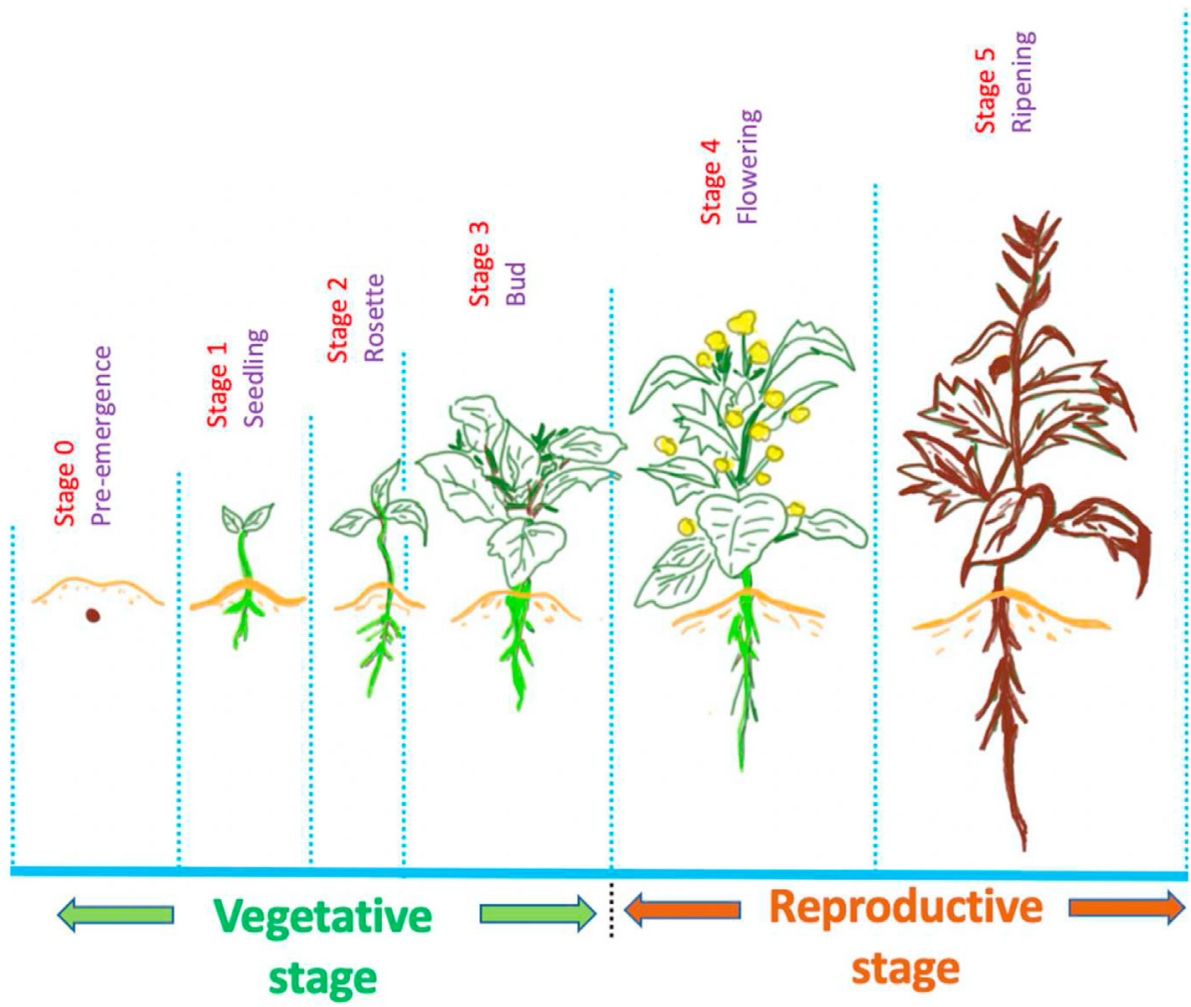
Vegetative and reproductive stages of plants including canola cultivars.

The vegetative and reproductive stages of canola plants are displayed in Figure 4 (inspired by [36]). For adequate canola seed imbibition (water absorption), the first step of germination confirms close contact between seeds and the moist soil that is low in inorganic salt and organic substances [33]. Several factors may influence the length of the growth stage, such as temperature, moisture, light, and nutrition [34]. Although canola is an agricultural cool season crop, temperatures below 5 °C can hinder plant growth, and extremely low temperatures may cause frost damage as the favorable temperature for canola plant growth is between 12 °C and 30 °C with an optimum temperature of 21 °C [35].

Once seed imbibition is successfully completed, a radicle root emerges from the seed, the root elongates downward to form a tap root, and root hairs help to anchor the development of the seed. Root growth is constant to 2 cm per day, and 4–15 days (d) after seeding, the seedling develops a short 1.25–2.5 cm stem [33], cotyledons emerge, then the first true leaf unfolds. Leaves are a major source of food for the growing plant as they are significant for photosynthates and required for growth; loss of leaves negatively impact the seed yield. At the bud stage, the stem elongates, and canola plants grow taller. However, at this stage flower buds remain enclosed by young leaves and the secondary branches may appear from axial buds of the upper leaves or from lower nodes. Flowering stage is signaled by the opening of the first yellow flowers on the terminal bud after 41–54 days from the germination stage and lasts between 14 to 21 days. The plants grow moderately during the flowering stage, but height of the plants stops at the peak of flowering. Seed filling is completed after 35–45 days of the flower opening and the firm green seeds at this stage contain the adequate level of oil and protein for future germination [33]. Seed ripening is the final stage of plant growth and development. The stems and seed pods gradually become brittle and dry at maturity. This stem biomass is used for producing canola fibres.

#### 4.1.2 Different observations on the effect of cultivars on stem physique

During the research study, the shortest heights of the canola plants were found to be 9ʺ, 13.5ʺ, 17ʺ, and 25ʺ for cultivars HYHEAR 1, Topas, 5440, and 45H29, respectively; the tallest plant heights were 40.25ʺ, 41.25ʺ, 38ʺ, and 50ʺ for HYHEAR 1, Topas, 5440, and 45H29, respectively. It was found that for all four cultivars, the tallest plants grew at the middle of the water bench, while the shortest plants grew at the back. This unequal growth pattern may have been due to the uneven distribution of sunlight during the growth stage.

Lodging (collapse of the stem when it can no longer support its own weight) is a significant problem that is visible during the growth stages of the canola plant [37] and can lead to an intensive loss of seed yield (%) [38]. Lodging resistance can be improved by introducing dwarf genes such as, ds-1 for canola [37] into cereal crops. There are many dwarf genes associated with canola plants, such as BnGID1, Bnrga-ds, BnaAnng13910D, BnaA09g53500D, Bra017367, and BnaA09g53470D [37, 39, 40, 41, 42, 43, 44, 45]. These dwarf genes, or a variation of such dwarf genes are possibly present in the canola cultivars used in this current research work, causing a variation of plant heights, although root length was consistent at 4.25ʺ for all cultivars.

#### 4.1.3 Non-parametric statistical analysis of canola stems of different canola cultivars

Chi-square (X^2^), a non-parametric statistical analysis test, was conducted to investigate the specific distribution of the samples i.e., 96 counts per cultivar (HYHEAR 1, Topas, 5440, 45H29) to analyze the effect of cultivar on plant stems. Four variables were chosen for this test while comparing the physical growth pattern of the four cultivars: stem height variation; lodging; presence of ruptured branches; and presence of non-linear stems. Observed (OC) and expected (EC) counts for these four variables are detailed in Tables 3 and 4 for chi-square statistical analysis.

**Table 3:** Observed counts (OC) of different parameters of four canola cultivars.

Observed counts (OC)					
Cultivars	Stem height variation	Lodging support	Presence of ruptured branch	Presence of non-linear stem	Total
HYHEAR 1	61	63	0	73	197
Topas	73	87	24	89	273
5440	20	21	0	21	62
45H29	16	17	41	5	79
**Total**	**170**	**188**	**65**	**188**	**611**

**Table 4:** Expected counts (EC) of different parameters of four canola cultivars.

Expected count					
Cultivars	Stem height variation	Lodging support	Presence of ruptured branch	Presence of non-linear stem	Total
HYHEAR 1	55	61	21	61	197
Topas	76	84	29	84	273
5440	17	19	7	24	62
45H29	22	24	8	24	79
**Total**	**170**	**188**	**65**	**188**	**611**

*p-*value = 0.00 (α = 0.05); X^2^ = 179.

Since *p-*value (probability) < 0.05 and chi-square X^2^ value was found too large (179), a null hypothesis is rejected and the alternative hypothesis is accepted; that is, there is a significant difference between the observed counts and expected counts. Hence, displayed by the Chi-square test of independence, there is a non-random relationship between the variations of stem properties observed and the four cultivars of canola. In other words, the cultivar affects the physical characteristics of plant stems.

#### 4.1.4 Risk assessment factors during handling canola plants

Previous studies have shown that canola plants are prone to different herbivorous insects that live on plant leaves, such as *Mamestra configurata*, *Plutella xylostella* (L.), aphids *Myzus persicae* (Sulzer) [13]. Insect attack has a detrimental effect on the plants by reducing the plant growth and its biomass production through the reduction of the photosynthetic area (removal of leaf), changing the carbohydrate balance (sap suckers or fruit eaters), and weakening of the plant's physical structure (stem borers) [13]. Many plants have inherent bio-chemical components that protect up to a certain level against bio-stresses or insect pests like aphids. For example, alfalfa (*Medicago sativa* L*.)* has alfalfa saponins that act as a secondary defense compound inside the host plant against bio-stresses, but are not effective against aphids [46]. In the current study necessary chemical treatments, liquid soap treatments, and sulphur dust treatments were used to eliminate the aphids, black flies, powdery mildew, and other bio-stresses.

**Figure 5:**
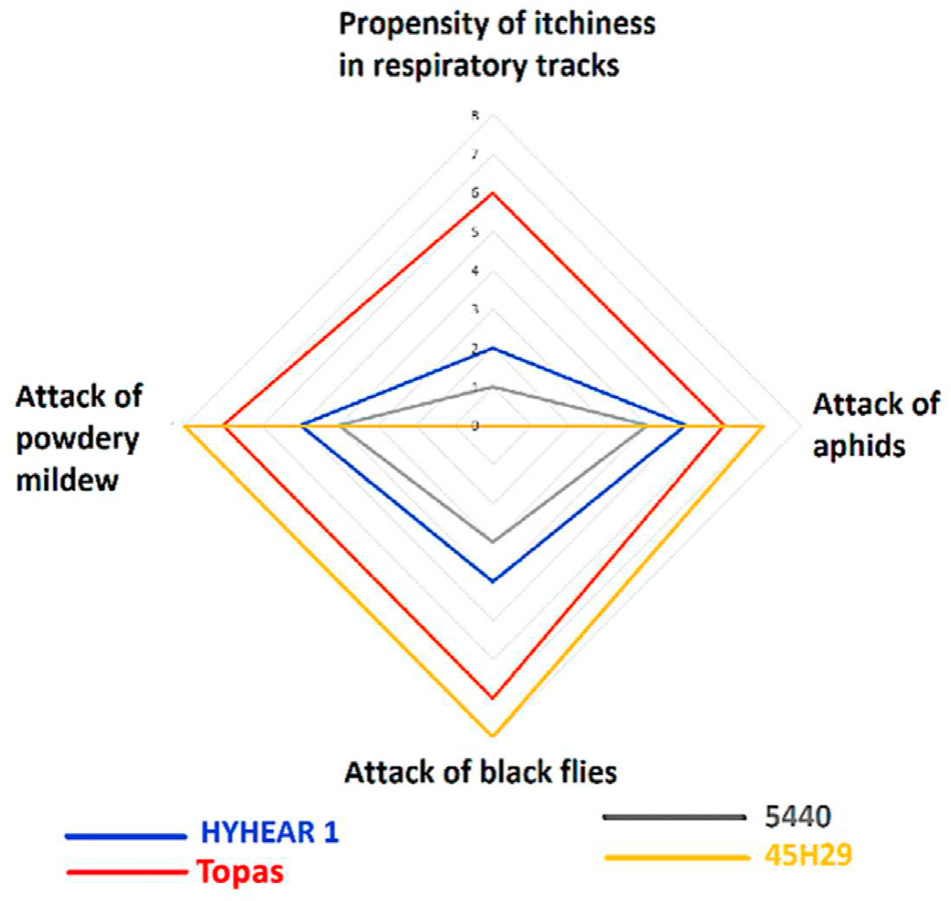
Risk assessment factors during handling of the canola cultivars.

The presence of these risk factor variables was different in intensity for each of the four cultivars. A radar chart (Figure 5) briefly compares (on a scale of 1–10, with 1 being lowest and 10 being highest) these risk factors among the four cultivars. The chart displays that the human respiratory tract is highly prone to the plant dusts of Topas but not towards 45H29. Plant dust from cultivar 45H29 caused no itchiness to face and body; Topas and HYHEAR 1 caused mild skin itchiness to face and hand: Topas more so than HYHEAR 1. Attack by aphids, black flies, and powdery mildew was most likely for cultivar 45H29, and least likely for cultivar 5440; Topas ranked second in vulnerability towards insect and mildew attack.

#### 4.1.5 Hygroscopic, thermal and different physical attributes of canola stems

Table 5 displays that HYHEAR 1 stems (9.99 ± 1.65%) had the highest moisture regain followed by 45H29 (9.96 ± 1.97%), while Topas stems demonstrated the lowest moisture regain propensity. However, no significant difference (*p* = 0.77 > 0.05) was present in the moisture regain characteristics of these four cultivars.

**Table 5:** Characteristics of virgin canola fibre, 10% softener treated canola fibre, and canola stems of four different canola cultivars.

Sl	Properties	N	HYHEAR 1	Topas	5440	45H29	CV,%	*p*
1	Moisture regain (%) of virgin fibre	3	12.53 ± 3.37^a^	9.38 ± 2.75^a^	13.82 ± 3.15^a^	13.24 ± 3.40^a^	14.46	0.59
2	Breaking load (lb) of virgin fibre	6	11.23 ± 2.77^a^	12.58 ± 3.09^a^	11.20 ± 1.61^a^	11.30 ± 3.09^a^	5.79	0.77
3	Strength index (lb/mg) of virgin fibre	6	1.99 ± 0.79^a^	2.48 ± 0.39^a^	1.81 ± 0.31^a^	1.77 ± 0.23^a^	16.11	0.07
4	Breaking tenacity (gf/tex) of virgin fibre	6	12.76 ± 5.05^a^	16.13 ± 2.57^a^	11.98 ± 2.07^a^	12.13 ± 1.57^a^	14.71	0.10
5	Tensile strength (MPa) of virgin fibre	6	168 ± 66.52^a^	215.30 ± 34.30^a^	162 ± 28.02^a^	169.95 ± 21.99^a^	13.74	0.13
6	Moisture regain (%) of treated fibres^z^	3	7.64 ± 0.03^a m n^	6.03 ± 0.05^a m s^	7.21 ± 0.06^am^	7.18 ± 0.06^ans^	9.83	0.00
7	Breaking load (lb) of treated fibres^z^	6	10.68 ± 3.40^a^	10.99 ± 1.98^a^	9.88 ± 1.79^a^	11.48 ± 2.53^a^	6.24	0.73
8	Strength index (lb/mg) of treated fibres^z^	6	1.28 ± 0.38^a m n^	1.93 ± 0.56^a m s^	1.41 ± 0.13^as^	1.84 ± 0.25^an^	19.71	0.01
9	Breaking tenacity (gf/tex) of treated fibres^z^	6	8.23 ± 2.47^a m n^	12.59 ± 3.66^a mq^	9.30 ± 0.84^aqs^	12.52 ± 1.73^ans^	20.93	0.01
10	Tensile strength (MPa) of treated fibres^z^	6	108.33 ± 32.49^amn^	167.98 ± 48.90^a m s^	125.74 ± 11.35^asq^	175.45 ± 24.25^anq^	22.51	0.01
11	Density (g/cc) of treated fibres^z^	7	1.34 ± 0.0009^a m^	1.36 ± 0.0007^a m^	1.38 ± 0.0007^am^	1.43 ± 0.0011^am^	2.80	0.00
12	Diameter range (μm) of treated fibres^z^	22	86.93 ± 57.12^a^	81.54 ± 31.78^a^	64.38 ± 26.22^a^	78.37 ± 47.79^a^	12.38	0.34
13	Minimum dia (μm) of treated fibres^z^	22	30.39	33.35	27.32	26.11		
14	Maximum diam (μm) of treated fibres^z^	22	213.60	146.67	109.25	207.33		
15	Thermal resistance (^o^C) of treated fibres^z^	3	242.50 ± 0.40 ^ag^	257.23 ± 0.51 ^ag^	249.33 ± 0.45 ^ag^	237.63 ± 0.51 ^ag^	3.45	0.00
16	Moisture regain (%) of stem	3	9.99 ± 1.65^a^	8.64 ± 1.12^a^	9.43 ± 2.18^a^	9.96 ± 1.97^a^	6.63	0.77
17	Thermal resistance (^o^C) of stem	3	262.5 ± 2.22 ^ag^	269.3 ± 2.25 ^ag^	250.3 ± 0.12 ^ag^	240.7 ± 2.10 ^ag^	5.05	0.00
18	Diameter range (mm) of stem	50	4.61 ± 0.77 ^ag^	4.92 ± 0.60^a^	5.01 ± 1.21^agh^	4.61 ± 0.91 ^ah^	4.37	0.04
19	Maximum dia (mm) of stem	50	6.24	5.98	8.95	6.99		
20	Minimum dia (mm) of stem	50	2.78	3.20	2.84	2.96		
21	L∗ (lightness/darkness) of stem	6	57.36 ± 4.72	60.83 ± 1.92	62.15 ± 1.17	65.98 ± 2.15		0.92
22	a∗ (red/green) of stem	6	6.89 ± 0.38	7.39 ± 0.78	6.14 ± 0.47	6.29 ± 0.63		0.73
23	b∗ (yellow/blue) of stem	6	19.30 ± 1.11	23.01 ± 1.90	21.13 ± 0.98	23.53 ± 2.06		0.71

^a^Mean ± Standard deviation.

^g, h, m, n, s, q^ Significant variation (at α = 0.05) between a pair after conducting Fisher's LSD test.

^z^ [4].

L∗ + = lighter; - = darker; a∗ (+ = redder; - = greener); b∗ + = yellow; - = bluer.

Investigation of the thermal resistance of the plant stems demonstrated significant variance (*p* = 0.00 < 0.05) among the stems of all the four cultivars (Table 5). Topas stems (269.3 ± 2.25 °C) showed the highest thermal resistance, whereas 45H29 stems (240.7 ± 2.10 °C) displayed the lowest among all the four cultivars.

Maximum diameter of plant stems was displayed by 5440 (8.95 mm), and the least by HYHEAR 1 (2.78 mm) (Table 5). Significant variation (*p* = 0.04 < 0.05) of plant stem diameters was present among the four cultivars, as shown by the chi-square test. Table 5 also displays that 5440 (5.01 ± 1.21 mm) showed the highest mean stem diameter; and HYHEAR 1 the lowest (4.61 ± 0.77 mm). The pairs showing significant variance between them were HYHEAR 1 and 5440; and 5440 and 45H29 (Table 5).

Table 5 displays no significant variance (*p* < 0.05) among the L∗, a∗, and b∗ values of the plant stems. The L∗ (57.36, 60.83, 62.15, 65.98), a∗ (6.89, 7.39, 6.14, 6.29), and b∗ (19.30, 23.01, 21.13, 23.53) values are quite close in value to each other and quite comparable for HYHEAR 1, Topas, 5440, and 45H29 cultivars, respectively, which may lead to the assumptions of similar reactivity towards treatment involved in bast-fibre dyeing and chemical finishing. The plant biomass that is collected, is typically dead stems, having no presence of green pigments, but rather a light brown color which is the combination of redness and yellowness (positive value of L∗, a∗, b∗). The value of L∗ (Table 5) is neither zero nor in the negative co-ordinates, not black in color.

### 4.2 Statistically identifying the influential parameters for optimum fibre yield

The bottom row of Table 6 displays the main effect and the average values of each design points. Table 7 displays the statistical significance (*p*-values) of this Ruggedness test, displaying the significant effects of four experimental design factors (D, A, C, and E) (*p* < 0.05). Orientation of stems (D) with respect to the water flow surface had the largest significant effect on the response followed by retting bath temperature (A), retting time (C), and stalk rotation frequency (E).

**Table 6:** Optimum fibre yield (%) Ruggedness test calculations.

Order	A	B	C	D	E	F	G	Rep 1	Rep 2	Rep Ave	Rep Diff
1	+1	+1	+1	-1	+1	-1	-1	8.94	9.46	9.20	-0.52
2	-1	+1	+1	+1	-1	+1	-1	9.80	9.97	9.89	-0.17
3	-1	-1	+1	+1	+1	-1	+1	10.98	11.64	11.31	-0.66
4	+1	-1	-1	+1	+1	+1	-1	11.45	12.08	11.77	-0.63
5	-1	+1	-1	-1	+1	+1	+1	6.20	6.80	6.50	-0.60
6	+1	-1	+1	-1	-1	+1	+1	8.80	8.10	8.45	0.70
7	+1	+1	-1	+1	-1	-1	+1	12.10	11.30	11.70	0.80
8	-1	-1	-1	-1	-1	-1	-1	2.10	2.30	2.20	-0.20
**Av+**	10.28	9.32	9.71	10.35	9.69	9.15	8.26				
**Av-**	7.47	8.43	8.04	6.59	8.06	8.60	8.26				
**Main effect**	2.81	0.89	1.67	3.77	1.64	0.55	0.00				

Standard deviation S_d_ = 0.59, Estimated std. deviation S_r_ = 0.42; Standard error effect S_effect_ = 0.21.

**Table 7:** Statistical significance of effects for test method for optimum fibre yield (%).

Effect order, e	Effect	Estimated effect	Student's t	*p*-value	
7	D	3.77	17.95	0.0000004^g^	<0.001
6	A	2.81	13.38	0.0000031^g^	<0.001
5	C	1.67	7.95	0.0000947^g^	<0.001
4	E	1.64	7.81	0.0001063^g^	<0.001
3	B	0.89	4.24	0.01	>0.001
2	F	0.55	2.62	0.03	>0.001
1	G	0	0	1.00	>0.001

g Significant at the 5% level; *p*-value is two-sided tail probability of Student's -t with 7 degrees of freedom.

### 4.3 Comparison among available surface modification methods for canola

**Figure 6:**
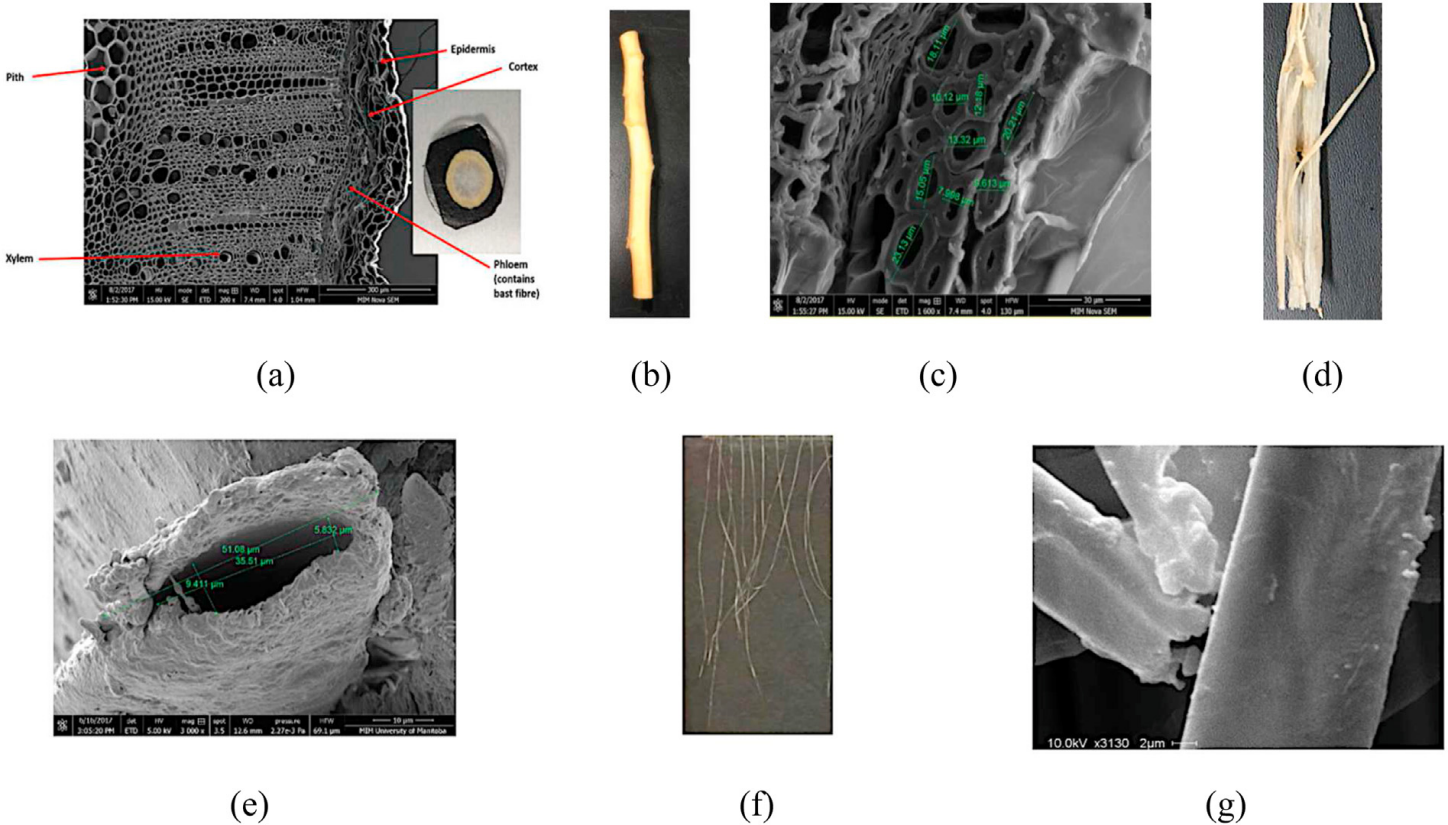
Cross-sectional view of canola (*Brassica napus* L.) plant stem with different fibre size and shape in its exterior (a: 200x, b); cluster of virgin fibres (c: 1600x, d) of different diameters (7.9 μm, 9.6 μm, 10.1 μm, 12.1 μm, 13.3 μm, 15.1 μm, 18.1 μm, 20.2 μm, 23.13 μm); and inherent hollow single canola fibres with smooth longitudinal surface (scaleless or twistless) contributing to their lightweight characteristics (e: 3000x, f, g: 3130x). SEMs reproduced with permission [4].

Canola fibres are found to be world's most light-weight bast-fibre (HYHEAR 1: 1.34 ± 0.0009 g/cc; Topas: 1.36 ± 0.0007 g/cc; 5440: 1.38 ± 0.0007 g/cc; 45H29: 1.43 ± 0.0011 g/cc) (Table 5) because of its hollow structure [4]. The cross-sectional view (Figure 6a) of the canola plant stem (Figure 6b) reveals the hollow structure of fibres (Figure 6e). Further, the fibre diameters vary both between and among the cultivars (HYHEAR 1: 86.93 ± 57.12 μm; Topas: 81.54 ± 31.78 μm; 5440: 64.38 ± 26.22 μm; 45H29: 78.37 ± 47.79 μm) (Table 5). Fibre individualization, which is essential for textile processing can be obtained by suitable surface modification techniques.

The SEM micrographs display two forms of canola fibres: (i) virgin fibres bundled together (Figures 6 c, d) before individualization; and (ii) single fibres (Figures 6 e, f, g) separated from the fibre bundle after surface modification. Canola fibres inherently exhibit the tendency of clinging together as displayed in Figure 6 (d) or in the SEM micrograph of Figure 6 (c). The SEM micrograph (Figure 6c) also reveals that single fibres of different diameters (7.9 μm, 9.6 μm, 10.1 μm, 12.1 μm, 13.3 μm, 15.1 μm, 18.1 μm, 20.2 μm, 23.13 μm) cluster together to form a fibre bundle. After surface modification, single fibres (Figure 6f) can be individualized from the fibre tuft using a fine needle and tweezers.

**Figure 7:**
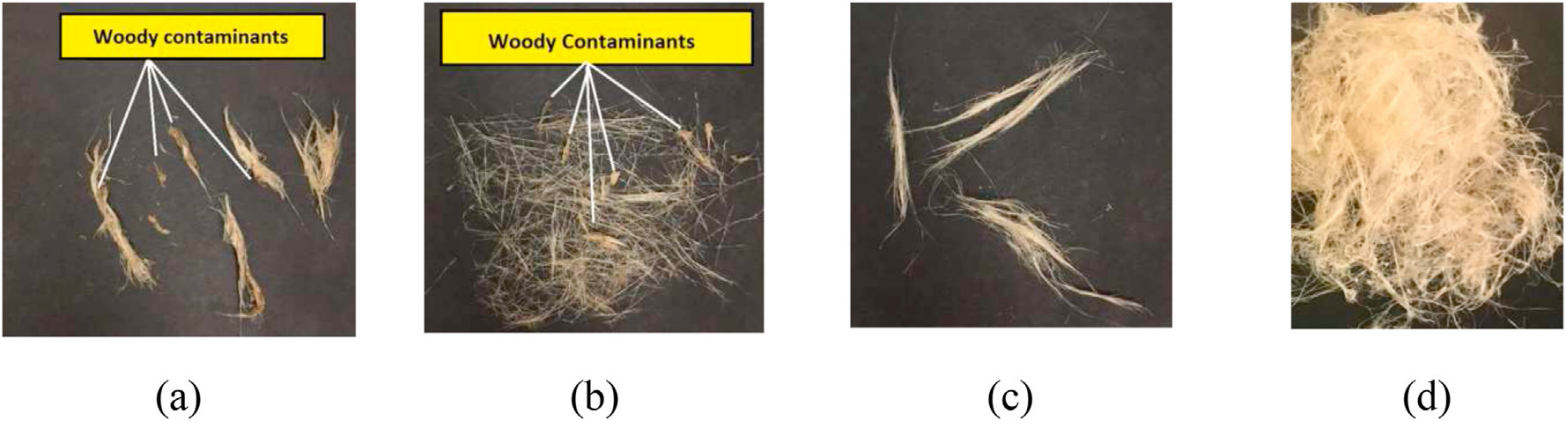
Canola fibres after treating with enzyme recipe (a), enhanced enzyme recipe (b), 3% softener plus enzyme recipe (c), and 10% softener recipe (d).

Figure 7 displays the canola fibres after treatment with enzyme recipe (Figure 7a), enhanced enzyme recipe (Figure 7b), 3% softener plus enzyme recipe (Figure 7c), and 10% softener recipe (Figure 7d). It can be seen that fibres still cling to woody contaminants after the enzyme and enhanced enzyme treatments (Figures 7 a, b), which degrade fibre quality or hamper subsequent fibre processing stages such as spinning. Further, both the enzymatic treatment and enhanced enzymatic treatments produced short fibres and caused fibre losses which contribute to lower the fibre yield (%). However, 3% softener (plus enzyme) and 10% softener treatment produced fibres with superior qualities of improved fibre individualization, flexibility, and softness (Figures 7 c, d). The treatment of 3% softener (plus enzyme) produced superior fibre quality, but poor fibre length. and reduced fibre yield (%), compared to 10% softener treatment.

**Figure 8:**
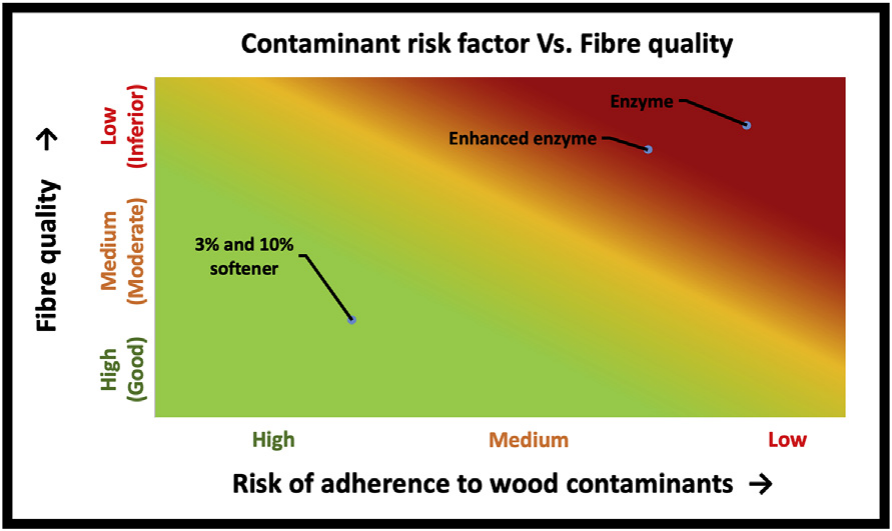
Impact of woody contaminants in degrading fibre quality.

Figure 8 is a risk assessment chart that shows a relationship between quality parameters (softness, individualization, and flexibility) of fibre and presence of woody contaminants. The more woody contaminants that cling to the fibre surface, the lower the fibre quality parameters because of increased fibre breakage, lower permeability to chemicals for surface modification, reduced process yield, lower fibre individualization possibility, greater fibre stiffness, and higher variability. Further, the presence of high woody contaminants increases processing time, manual labour, and cost of operation.

**Figure 9:**
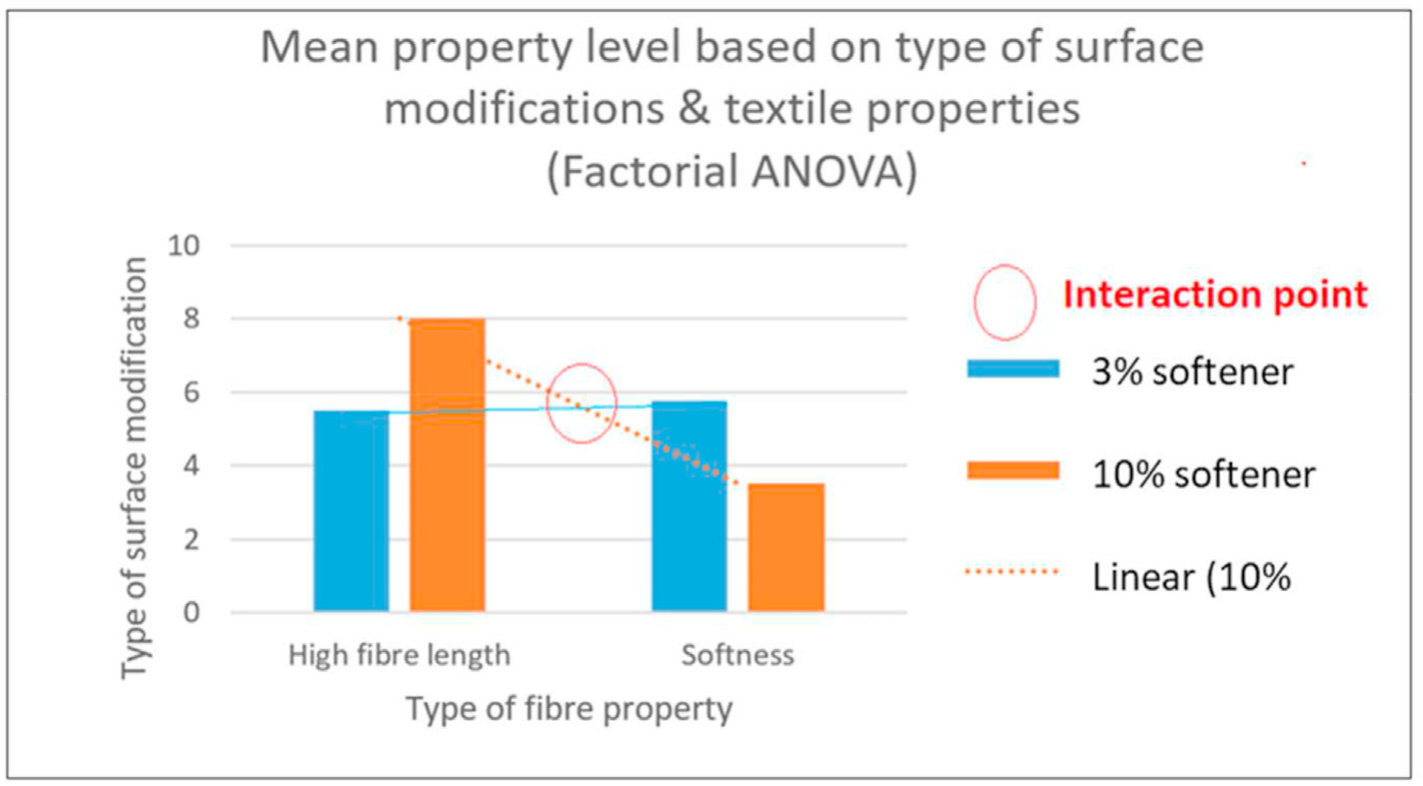
Mean property level (softness, fibre length) based on type of surface modifications and bast-fibre properties constructed by factorial ANOVA.

The factorial ANOVA (Table 8) outlines the relationship between main effects and interaction levels (Figure 9), where the property types and surface modification types were independent variables (IVs) and property level was the dependent variable (DV). The mean score of (higher degree of) fibre length was 5.5 (on a scale of 1–10) for 3% softener (plus enzyme treatment) and 3.5 is the mean score of softness level (on a scale of 1–10) when treated by 10% softener and so on. Mean of means at the bottom row (6.75, 4.625) shows the effect of type of bast-fibre properties on the property level. It can be seen that (6.75 > 4.625), fibres with high fibre length property has a better bast-fibre property level than fibres with lower fibre length. However, there is no main effect for the types of chemicals (surface modification) used since the mean of means (5.625, 5.75) are quite close to each other.

**Table 8:** Factorial ANOVA to investigate the main effects and interaction level of canola fibre properties.

		Type of bast-fibre properties (mean)		
		Fibre length	Fibre Softness	Mean of means
Type of surface modification	3% softener plus enzyme	5.5	5.75	5.625
	10% softener treatment	8	3.5	5.75
	**Mean of means**	6.75	4.625

As seen in Figure 9, the intersection of the bar graph trend lines shows interaction effects of the surface modification techniques on fibre length and softness. The nature of this interaction effect can be stated as: when higher fibre length is needed, 10% softener treatment is more effective than 3% softener (plus enzyme) treatment. However, the nature of this interaction demonstrates an inverse relationship for the level of fibre softness, as 10% softener treatment produces higher fibre length, whereas 3% softener (plus enzyme) treatment produces higher fibre softness. Consequently, 10% softener treated fibres were found suitable for further fibre characterization, which has been investigated in our previous research works [4] and the resultant fibre properties are reported in Table 5.

### 4.4 Comparative analysis between virgin and chemically modified fibre properties

Table 5 compares the hygroscopic and mechanical properties of raw water retted virgin canola fibres (which were never treated with any kinds of chemicals or enzymes) with chemically treated (10% softener treated) canola fibres. It can be seen that there is no significant variation (*p* > 0.05) among the water-retted virgin fibres of the four cultivars regarding their hygroscopic and mechanical properties, such as moisture regain, breaking load, strength index, breaking tenacity, and tensile strength. However, the case was totally different for the chemically treated fibres of these same four cultivars (Table 5). The co-efficient of variation (CV%) was found least regarding the breaking load (5.79%) and highest regarding strength index (16.11%) among the water-retted virgin fibres of the four cultivars (Table 5). For chemically treated fibres, breaking load (6.24%) exhibited the lowest CV% and tensile strength (22.51%) the highest (Table 5). In moisture regain, 5440 (13.82 ± 3.40) exhibited the highest, and HYHEAR 1 (12.51 ± 3.37%) the lowest. Surprisingly, after the chemical treatment, the softener treated fibres for each of the four cultivars demonstrated a lower moisture regain compared to its virgin counterpart (Table 5). A separate research work revealed that moisture regain of cellulosic fibre is reduced when treated with a softener [47], as was verified in this study using canola fibre.

Further, from Table 5 it was found that virgin Topas fibres demonstrated superior breaking load (12.58 ± 3.09 lb), strength index (2.48 ± 0.39 lb/mg), breaking tenacity (16.13 ± 2.57 gf/tex), and tensile strength (215.30 ± 34.30 MPa) among all the four cultivars: 5440 exhibited the lowest breaking load (11.20 ± 1.61 lb), tenacity (11.98 ± 2.07 gf/tex), tensile strength (162 ± 28.02 MPa) and 45H29 the lowest strength index (1.77 ± 0.23 lb/mg) among all the virgin fibres. Among the chemically treated fibres, 5440 (9.88 ± 1.79 lb) again exhibited the lowest breaking load and 45H29 (11.48 ± 2.53 lb) the highest; HYHEAR 1 (1.28 ± 0.38 lb/mg) exhibited the lowest strength index and Topas (1.93 ± 0.56 lb/mg) the highest; HYHEAR 1 (8.23 ± 2.47 gf/tex) exhibited the lowest breaking tenacity and Topas (12.59 ± 3.66 gf/tex) the highest; and 5440 (167.98 ± 48.90 MPa) exhibited the lowest tensile strength and 45H29 (175.45 ± 24.25 MPa) the highest.

It was interesting to discover that after surface modification, the mechanical properties of the cultivars were lowered, except for 45H29 that demonstrated an increase of 1.59% in breaking load (lb), 3.95% in strength index (lb/mg), 3.22% in breaking tenacity (gf/tex), and 3.24% in tensile strength (MPa); however, moisture regain (%) of this cultivar was reduced by 45.77% after surface modification. Moisture regain, breaking load, strength index, breaking tenacity, and tensile strength all decreased for HYHEAR 1, Topas, and 5440.

**Figure 10:**
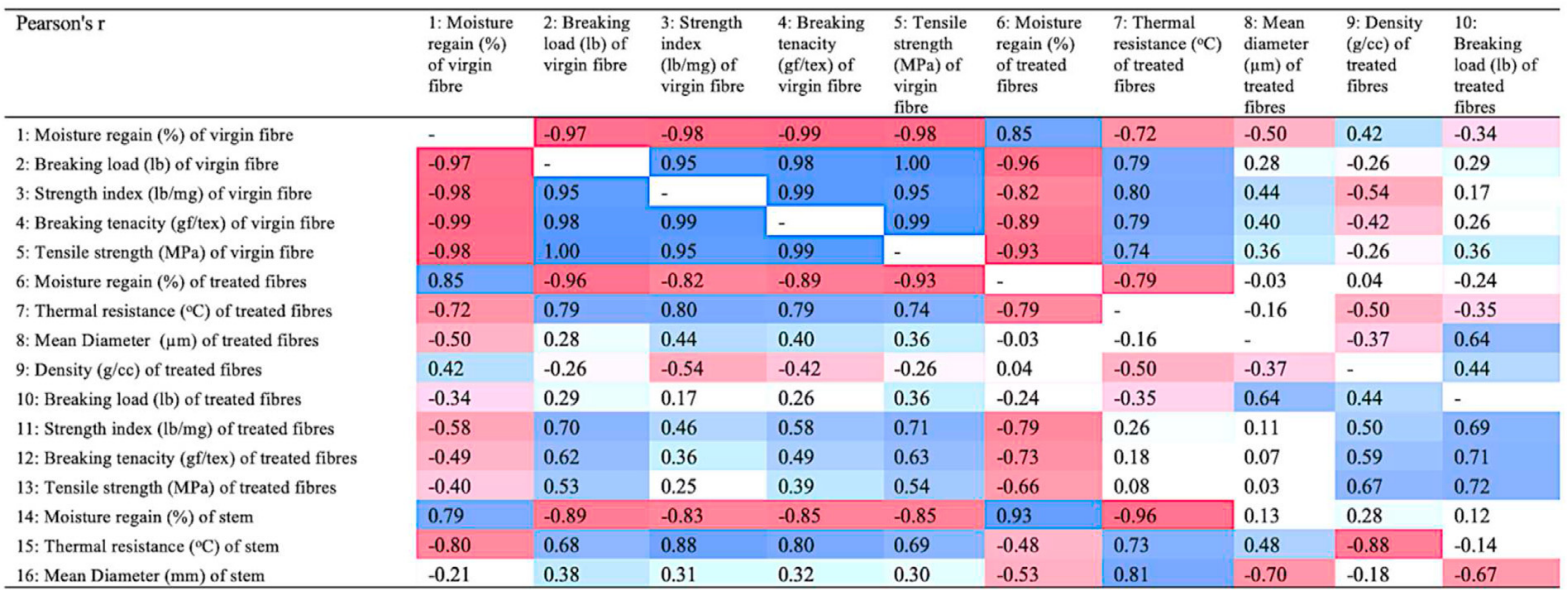
Correlation (Pearson's r) matrix among the different variables of canola (darker the redness, more negative the correlation and vice-versa; darker the blueness, higher the positive correlation and vice-versa).

Pearson's correlation coefficient (r) (Figure 10) exhibits negative correlation coefficients of -0.97, -0.98, -0.99, -0.98 between moisture regain of virgin fibres and breaking load, strength index, breaking tenacity, and tensile strength, respectively. Further, the correlations between density and breaking load or moisture regain and diameter are 0.44 and 0.04, respectively. As with virgin fibres, the mechanical properties of modified fibre also exhibited negative correlation between moisture regain and breaking load, and moisture regain and tensile strength. From the correlation matrix, it is seen that the mechanical properties of both virgin and treated (chemically modified) fibres are positively co-related and that thermal resistance is negatively correlated to hygroscopic properties of canola stems and fibres.

**Figure 11:**
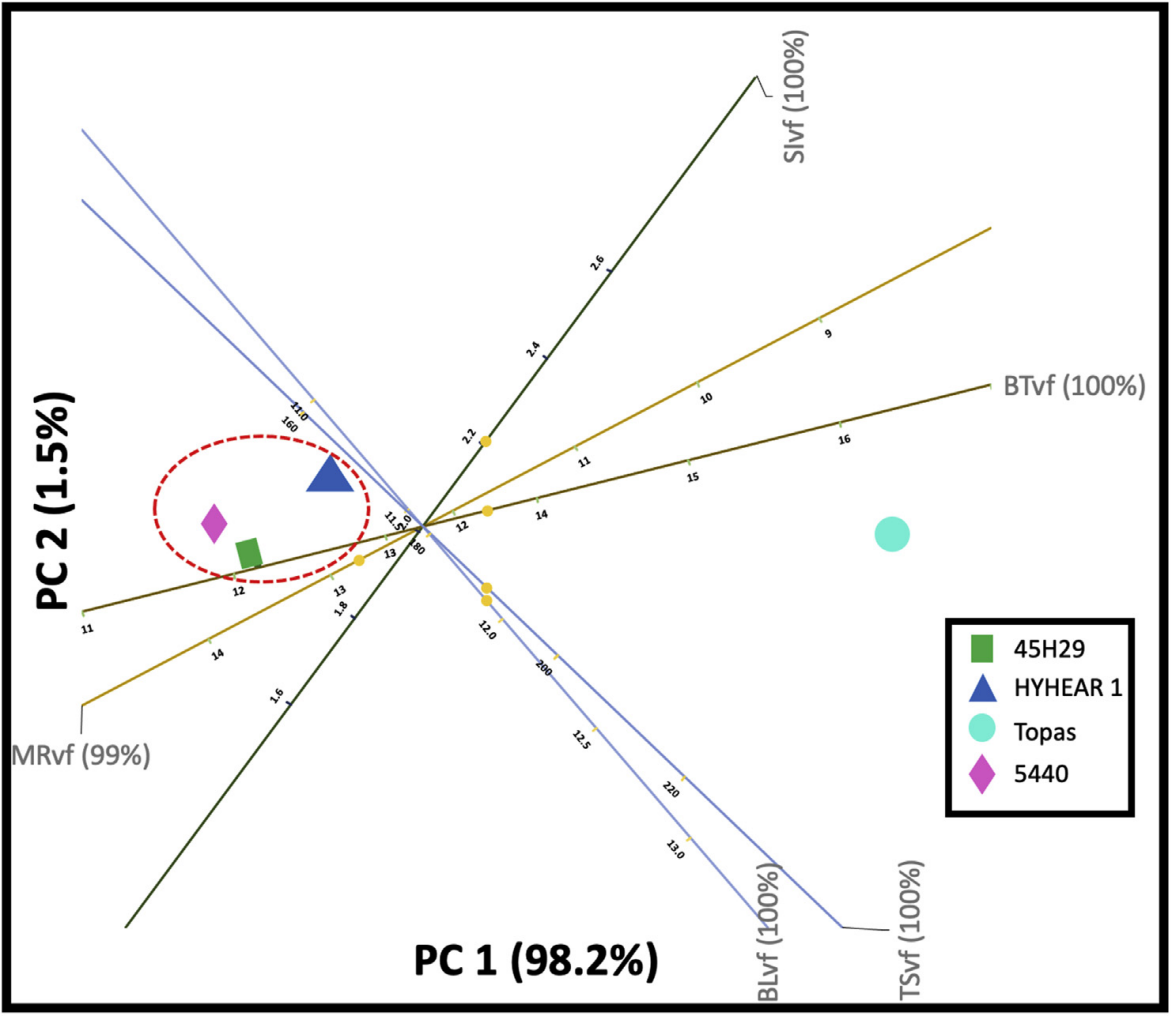
Principal component analysis (PCA) biplot for virgin fibres of canola (SI: strength index; BT: breaking tenacity; TS: tensile strength; BL: breaking load; MR: moisture regain; vf: virgin fibres of canola).

**Figure 12:**
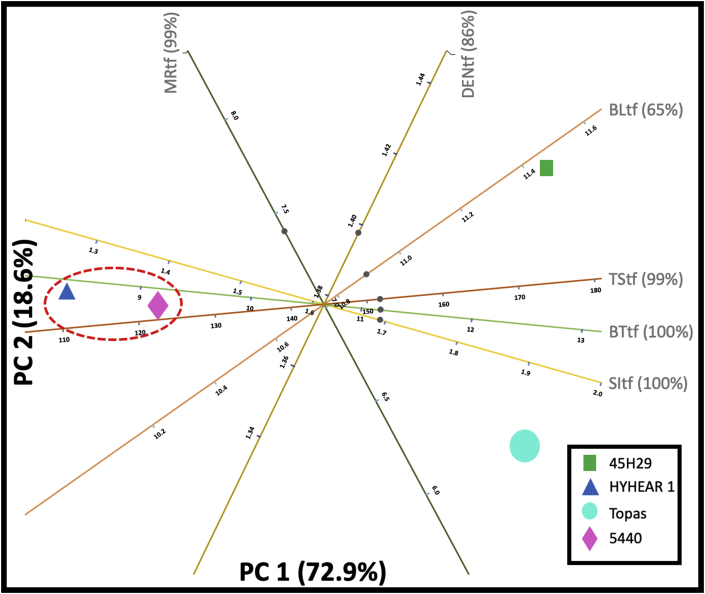
Principal component analysis (PCA) biplot for treated fibres of canola (SI: strength index; BT: breaking tenacity; TS: tensile strength; BL: breaking load; MR: moisture regain; DEN: density; tf: treated (chemically modified) canola fibres).

A biplot shows the 2D approximation to the original multi-dimensional space for a given dataset. Figures 11 and 12 represent 2D PCA biplots, where each point represents the cultivars, and the axes represent the variables. In these 2D biplots, the closeness of points shows that the corresponding cultivars have similar profiles, and the points further away, dissimilar profiles. Any points on the plot can be projected orthogonally on the axes to show the approximate value of the variable. For instance, the predicted values (12.70%, 11.20 lb, 1.90 lb/mg, 12.70 gf/tex, 169 MPa) (Figure 11) for the moisture regain (%), breaking load (lb), strength index (lb/mg), tenacity (gf/tex), and tensile strength (MPa) of virgin canola are fairly close to their true values (12.53%, 11.23 lb, 1.99 lb/mg, 12.76 gf/tex, 168) (Table 5). The case was also found identical for the chemically treated fibres (predicted values: 6.5%, 10.9 lb, 1.92 lb/mg, 12.7 gf/tex, 168 MPa, 1.36 g/cc; true values: 7.18%, 11.48 lb/mg, 1.84 lb/mg, 12.52 gf/tex, 175.45 MPa, 1.43 g/cc) for moisture regain (%), breaking load (lb), strength index (lb/mg), tenacity (gf/tex), tensile strength (MPa), density (g/cc), and diameter (μm) (Figure 12) (Table 5). Further, Figure 11 displays that 99% and 100% of the variation in the original variables (of moisture regain, breaking load, strength index, tenacity, tensile strength) are explained by the principal component 1 (PC1) and principal component 2 (PC2) in the multivariate space. PC1 and PC2 also explain 99%, 86%, 65%, 99%, and 100% (Figure 12) of the variation in the original variables for moisture regain, density, breaking load, tensile strength, breaking tenacity, and strength index, respectively. Breaking tenacity and moisture regain are the most important factors accounting for PC1 in Figure 11, although in opposite directions. For Figure 12, tensile strength and breaking tenacity have similar effects as strength index and are the most important factors accounting for PC1, whereas density contributes to PC2.

The results of 2D PCA biplot for virgin fibres (Figure 11) represents 99.7% of the variance of the original dataset and indicate that the controlled cultivar Topas and experimental cultivars (HYHEAR 1, 5440, 45H29) are graphically separated along the projected plane, which infers that the controlled cultivar Topas is different from the experimental cultivars before any chemical or enzymatic modification. It can also be seen that Topas is superior regarding breaking tenacity and inferior in moisture regain from the virgin fibres of the other three cultivars HYHEAR 1, 5440, and 45H29. To understand the interpretation of the biplot result, it can be hypothesized that PC1 is displayed in X-axis and PC2 in Y-axis. Focusing on PC1 first, breaking tenacity and moisture regain are on opposite ends, which infers that cultivars exhibiting a higher degree of moisture regain may exhibit lower breaking tenacity and vice-versa, which was also found in the research study (Table 5). For example, cultivars with higher moisture regain (from high to low: 5440 > 45H29 > HYHEAR 1 > Topas) tend to exhibit lower breaking tenacity (from low to high: 5440 < 45H29 < HYHEAR 1 < Topas) (Table 5). Apparently, breaking load and tensile strength of cultivars sit closer to each other, and their variations are better described in PC2. Hence, it can be seen that the vectors or axes of the variables show the directions of the PC (principal component) loadings and a dot represents an individual canola cultivar. The higher the score of a dot (cultivar) towards the direction of a PC, the stronger it correlates to that PC and vice-versa. It can be seen that breaking tenacity of Topas is located towards the direction of PC1 and closer to PC1; whereas, the HYHEAR 1, 5440, and 45H29 sit in the opposite direction and closer to PC1. Hence, the higher the PC1, the higher would be the breaking tenacity of Topas compared to HYHEAR 1, 5440, and 45H29, as can be verified in Table 5. Similarly, moisture regain of HYHEAR 1, 5440, and 45H29 closer to PC1. A higher PC1 indicates a higher moisture regain for HYHEAR 1, 5440, 45H29 compared to Topas. Further, the biplot (Figure 11) displays that the experimental cultivars (HYHEAR 1, 5440, 45H29) are close to each other, forming a cluster representing similarity.

In a similar manner, the 2D PCA biplot for chemically modified fibres (Figure 12) represents 91.5% of the variance of the original dataset and indicates that the experimental cultivars HYHEAR 1 and 5440 form a cluster since they are close to each other. For all four cultivars, it can be seen that the mechanical properties (tensile strength, breaking tenacity, strength index) are close to each other, and closer to PC1. Both Topas and 45H29 are in the right quadrants, and the cultivars HYHEAR 1 and 5440 are in the left quadrants indicating stronger mechanical properties for Topas and 45H29 compared to HYHEAR 1 and 5440, as shown in Table 5. Further, 45H29 has a stronger profile in breaking load and Topas in strength index when compared to HYHEAR 1 and 5440. 45H29 is the outlier in Figure 12, which was observed prior to surface modification (Figure 11). As a result, 45H29 will behave differently than the other two experimental cultivars, which was also illustrated in the correlation matrix. Surface modification has improved the mechanical properties (of 45H29), but reduced the mechanical properties of HYHEAR 1 and 5440 creating variability, as discussed earlier.

**Figure 13:**
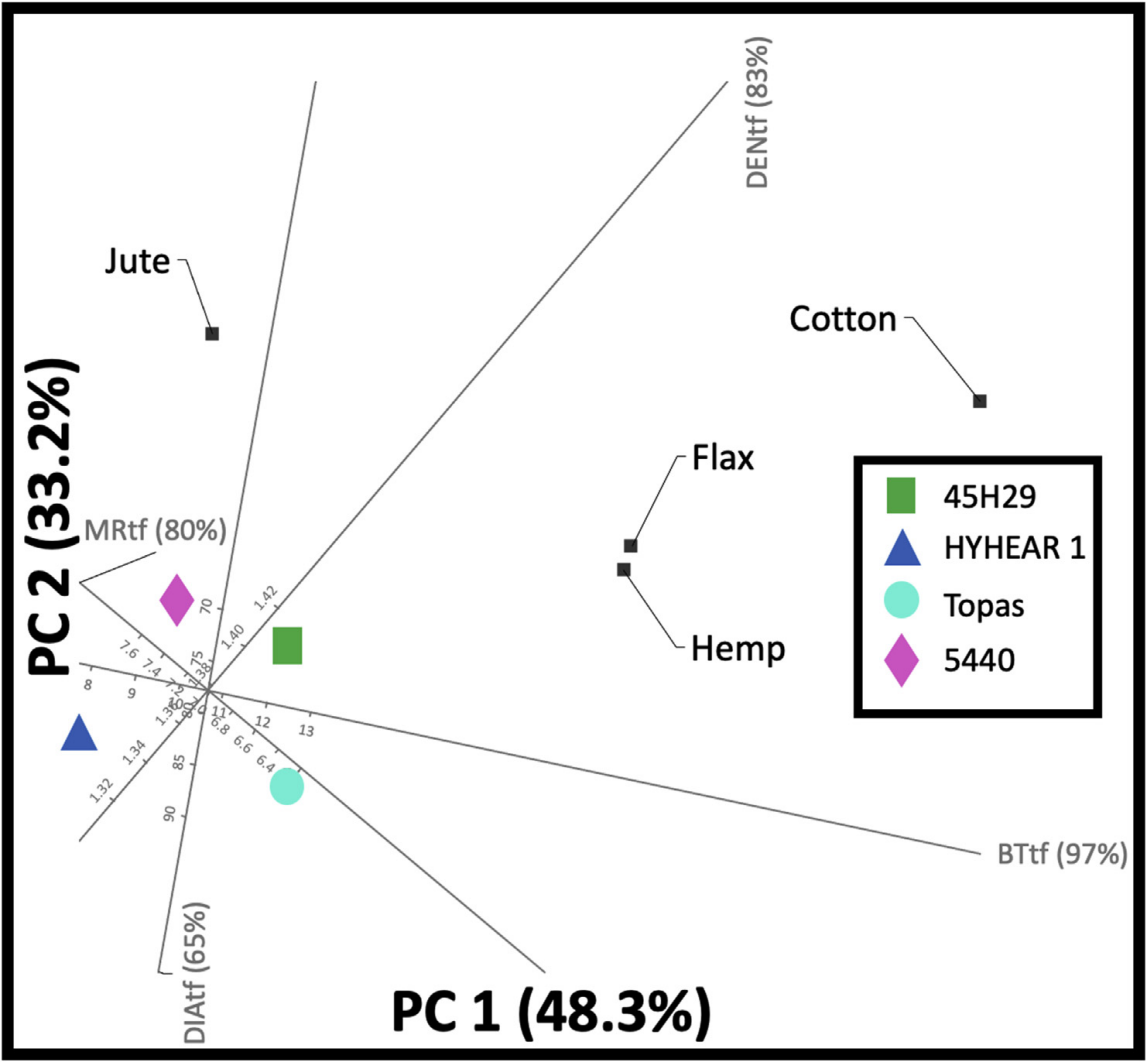
Principal component analysis (PCA) biplot to approximate the proximity of canola cultivars for wearable fibre application for blending with cotton or an alternative source to jute, hemp, flax for technical fibre application.

Such cluster formation created through 2D PCA biplots is effective for material engineers and fibre chemists to make informed decisions and devise action plans accordingly. For instance, the need of individual treatment processes or bast-fibre modification processes may be reduced since the engineers and scientists can save time, effort, and capital by segregating the canola cultivars in clusters and using the suggested cultivar based on the requirements for the end use application by defining an ideal point. Four ideal points of cotton, jute, hemp, flax fibre quality have been generated in Figure 13 to identify the proximity of the clusters of canola cultivars towards these ideal points. The results of 2D PCA biplot (Figure 13) represent that cultivar 5440 is in a better condition with respect to fibre properties of jute (moisture regain: 12%; density: 1.54 g/cc; dia: 25–200 μm; breaking tenacity: 18–56.70 g-force/tex) and 45H29 to hemp (moisture regain: 8%; density: 1.48 g/cc; dia: 25–100 μm; breaking tenacity: 18–56.70 g-force/tex) or flax (moisture regain: 12%; density: 1.54 g/cc; dia: 25–100 μm; breaking tenacity: 27-63 g-force/tex) for consumer wearables, or nonwoven or composite applications since all of these are bast fibres [7, 48]. Since the variation with cotton (moisture regain: 8.5%; density: 1.54 g/cc; dia: 38 μm; breaking tenacity: 41.30 g-force/tex) is high, the probability of using 45H29 and Topas as a blend in yarn spinning may be explored after rigorous research work [7, 48]. These clusters in 2D PCA biplots can be helpful in devising experimental designs to use canola fibres as an alternative source to available bast fibres for fibre reinforcements or nonwoven applications, or as a sustainable cellulosic source to blend with major cellulosic fibres (with minor adjustments), and may reduce the need of individual action plans for each canola cultivar as a consequence of reduced variability.

## 5 Conclusion

The holistic approach of this current research work represents a comprehensive and validated framework to support decision making modelling for end-use application of four different canola cultivars: HYHEAR 1, Topas, 5440, 45H29. This decision-support validated framework can be effective for material or technical industries to formulate necessary production plans based on the behavioral pattern of different canola cultivars to meet the end use requirement, therefore saving time, efforts and resources. Simultaneous and separate analytics of in-situ (virgin untreated fibres) and in-vivo (chemically modified) canola fibres also provide a decision support framework to the wearable fibre and composite experts. The current study emphasizes statistically analysis of observations collected from each processing stage, such as plant production, biomass collection, retting bath parameters during stem retting, chemical-enzymatic surface modifications of fibres, and drastic changes of hygroscopic and mechanical properties of virgin and chemically treated fibres for a comprehensive understanding to efficiently practice a more sustainable and cleaner production process of cellulosic lightweight bast-fibre from canola biomass.

## Declarations

### Author contribution statement

Ikra Iftekhar Shuvo: Conceived and designed the experiments; Performed the experiments; Analyzed and interpreted the data; Contributed reagents, materials, analysis tools or data; Wrote the paper.

### Funding statement

This research did not receive any specific grant from funding agencies in the public, commercial, or not-for-profit sectors.

### Data availability statement

Data included in article/supplementary material/referenced in article.

### Declaration of interests statement

The authors declare no conflict of interest.

### Additional information

No additional information is available for this paper.
